# Are low and middle-income countries prioritising high-value healthcare interventions?

**DOI:** 10.1136/bmjgh-2019-001850

**Published:** 2020-02-04

**Authors:** Ashley A Leech, David D Kim, Joshua T Cohen, Peter J Neumann

**Affiliations:** 1 Department of Health Policy, Vanderbilt University School of Medicine, Nashville, Tennessee, USA; 2 Center for the Evaluation of Value and Risk in Health, Institute for Clinical Research and Health Policy Studies, Tufts Medical Center, Boston, Massachusetts, USA

**Keywords:** health economics, health policy

## Abstract

**Introduction:**

Since resources are finite, investing in services that produce the highest health gain ‘return on investment’ is critical. We assessed the extent to which low and middle-income countries (LMIC) have included cost-saving interventions in their national strategic health plans.

**Methods:**

We used the Tufts Medical Center Global Health Cost-Effectiveness Analysis Registry, an open-source database of English-language cost-per-disability-adjusted life year (DALY) studies, to identify analyses published in the last 10 years (2008–2017) of cost-saving health interventions in LMICs. To assess whether countries prioritised cost-saving interventions within their latest national health strategic plans, we identified 10 countries, all in sub-Saharan Africa, with the highest measures on the global burden of disease scale and reviewed their national health priority plans.

**Results:**

We identified 392 studies (63%) targeting LMICs that reported 3315 cost-per-DALY ratios, of which 207 ratios (6%) represented interventions reported to be cost saving. Over half (53%) of these targeted sub-Saharan Africa. For the 10 countries we investigated in sub-Saharan Africa, 58% (79/137) of cost-saving interventions correspond with priorities identified in country plans. Alignment ranged from 95% (21/22 prioritised cost-saving ratios) in South Africa to 17% (2/12 prioritised cost-saving ratios) in Cameroon. Human papillomavirus vaccination was a noted priority in 70% (7/10) of national health prioritisation plans, while 40% (4/10) of countries explicitly included prenatal serological screening for syphilis. HIV prevention and treatment were stated priorities in most country health plans, whereas 40% (2/5) of countries principally outlined efforts for lymphatic filariasis. From our sample of 45 unique interventions, 36% of interventions (16/45) included costs associated directly with the implementation of the intervention.

**Conclusion:**

Our findings indicate substantial variation across country and disease area in incorporating economic evidence into national health priority plans in a sample of sub-Saharan African countries. To make health economic data more salient, the authors of cost-effectiveness analyses must do more to reflect implementation costs and other factors that could limit healthcare delivery.

**Key questions**

**What is already known?**

- Cost-effectiveness analyses can help decision makers maximise health gains per dollar spent on care, a tool that is particularly helpful amid restricted budgets.
- Several initiatives encourage integration of economic evaluation into countries’ prioritisation planning.

**What are the new findings?**

- Our findings indicate substantial variation across country and disease area in incorporating economic evidence into national health priority plans in sub-Saharan Africa.
- Most countries prioritised cost-saving interventions for human papillomavirus and HIV prevention and treatment; however, country prioritisation plans either omitted or were ambiguous regarding other cost-saving intervention areas such as antenatal syphilis screening and efforts towards specific neglected tropical diseases.

**What do the new findings imply?**

- Operational costs and other contextual factors may play a larger role than economic evidence in decision-making. It is therefore important that authors of cost-effectiveness analyses incorporate implementation and other contextual constraints into their evaluations that could limit healthcare delivery.
- Our list of cost-saving interventions can provide a good starting point for policymakers setting healthcare prioritisation plans for low and middle-income countries.

## Introduction

The 2030 Sustainable Development Goals call for greater access to affordable medications and interventions.^1 2^ Since its launch, many low and middle-income countries (LMIC) have made progress towards provision of universal healthcare coverage by prioritising access to essential services.^3–12^ That progress, however, has been slow.^10 13^ Health priority plans, sometimes referred to as ‘essential package of health services’ or ‘health benefit packages’, outline services that a government is providing or aspires to provide.^14–17^ Because resources are scarce, investing in ‘high-value’ health services, that is, those that produce the highest health gain ‘return on investment’, is critical.

Several initiatives encourage integration of economic evaluation into countries’ prioritisation planning.^3 6 9 11 18–21^ The Disease Control Priorities Network, for instance, proposes a ‘model’ benefits package that includes a subset of high-priority interventions (ie, interventions <$200/disability-adjusted life years (DALY) averted for low-income countries and <$500/DALY for lower middle-income countries).^20 22^

Our paper assesses whether LMICs have included ‘cost-saving’ health interventions in national health priority plans—that is, interventions that are projected to both improve population health and reduce costs by, for example, preventing expensive, downstream adverse health events. The main purpose of our study centres on the importance of using economic evidence in country priority setting.

## Methods

### Global Health Cost-Effectiveness Analysis Registry

The Tufts Medical Center Global Health Cost-Effectiveness Analysis (GHCEA) Registry, sponsored by the Bill and Melinda Gates Foundation, is a comprehensive database of English-language peer-reviewed cost-effectiveness analyses (CEA) containing 620 cost-per-DALY studies published through 2017 (updates are ongoing) (see the online supplementary appendix for a detailed overview of the GHCEA Registry). Of these 620 studies, we included CEAs applied to LMICs and published in the last 10 years (2008–2017) to reflect the latest medical interventions (table 1). We further classified cost-saving interventions by global burden of disease categories and reported the target country/region, target population, study perspective and time horizon (online supplementary appendix table 1).

10.1136/bmjgh-2019-001850.supp1 Supplementary data

**Table 1:** Incremental cost-effectiveness ratio and article characteristics among LMICs (2008–2017)

Characteristic	ICERs n (%)	Articles n (%)
Cost saving*	207 (6)	33 (9)
<$100/DALY	844 (25)	117 (30)
$100–$1000/DALY	1206 (36)	144 (37)
$1001–$10 000/DALY	739 (22)	66 (17)
>$10 000/DALY	218 (7)	21 (5)
Dominated	86 (3)	5 (1)
Total	3315 (missing=15)	392 (missing=6)

*Interventions that are cost saving and health improving.

DALY, disability-adjusted life year; ICER, incremental cost-effectiveness ratio; LMIC, low and middle-income country.

### Assessment of countries’ health priorities and cost-saving interventions

To assess whether LMICs prioritised cost-saving interventions within their latest national health strategic plans, we identified 10 countries with the highest global burden of disease^23^ and reviewed their health priority plans by gathering information on country government websites (eg, Federal Ministries of Health) and from US agencies (eg, US Agency for International Development). In cases where countries had additional disease-specific health plans, we also reviewed these (online supplementary appendix table 2). The 10 countries identified included: Cameroon, Lesotho, Liberia, Malawi, Nigeria, Sierra Leone, South Africa, Swaziland, Zambia and Zimbabwe.

We excluded countries for which we could not obtain health priority plans in English. We also omitted countries for which there were no reported cost-saving interventions (we excluded a total of 17 countries, listed in online supplementary appendix table 3).^23^ Next, we grouped all cost-saving interventions for each identified country. In cases in which studies provided only one cost-saving estimate and/or health gain estimate across multiple countries, we listed the intervention for each relevant country. For instance, there were five cost-saving interventions pertaining specifically to Malawi; however, there were 11 additional interventions that pertained to multiple countries that also included Malawi. We therefore included all 16 cost-saving interventions for Malawi and did the same for the other countries in our sample (online supplementary appendix table 2).

While the GHCEA Registry reflects data at the intervention level (eg, a specific test for prenatal syphilis screening), country policy documentation typically does not contain this level of detail. Therefore, we grouped each cost-saving intervention into a more general intervention category (eg, syphilis screening within antenatal visits) (online supplementary appendix table 2) and examined whether country health priority plans covered these broader health service areas. This approach was necessary to match our intervention-level data with less granular descriptions in each country’s strategic health plan. For each cost-saving intervention area, we designated one of the following findings for each country: (1) available policy documentation matches cost-saving intervention area, (2) cost-saving intervention area unspecified in policy documentation, or (3) intervention area not included in available policy documentation.

We designated the ‘unspecified’ finding in cases in which an intervention area might be broadly implied but unspecified. For example, Zambia’s Health Strategic Plan (2017–2021) outlines strategies for reducing the incidence of sexually transmitted infections. It also outlines a goal of strengthening services for antenatal care.^24^ However, it does not explicitly mention prenatal serological screening for syphilis, which is one of the cost-saving intervention areas in the Tufts GHCEA Registry. When we report on concordance, we accounted for studies only under the first category,^1^ that is, available policy documentation matches cost-saving intervention area.

### Patient and public involvement

Patients nor the general public were involved in the design of this study.

## Results

### Cost-saving interventions in LMICs

We identified 392 studies (63%) targeting LMICs in the last 10 years that reported 3315 cost-per-DALY ratios, of which 207 ratios (6%) represented interventions reported to be cost saving (table 2). Over half (53%) of the studies targeted interventions in sub-Saharan Africa. Communicable, maternal, neonatal and nutritional disorders made up the highest proportion (61%) of cost-saving interventions, with HIV and tuberculosis comprising nearly half (46%) of interventions within this category. Neglected tropical diseases and malaria comprised the second highest proportion (19%) within the communicable disease category. Of the cost-saving interventions that targeted non-communicable diseases, over three-quarters focused on neoplasms (59%) and mental and other behavioural disorders (28%) (table 3).

**Table 2:** LMIC GDP threshold-based categories (2008–2017)

Incremental cost-effectiveness ratio categories	Cost saving	<1×GDP	1–3×GDP	>3×GDP	Dominated	Total
Cost saving	207	0	0	0	0	207
<$100/DALY	0	844	0	0	0	844
$100–$1000/DALY	0	1204	2	0	0	1206
$1001–$10 000/DALY	0	648	77	14	0	739
>$10 000/DALY	0	78	66	74	0	218
Dominated	0	0	0	0	86	86
Total	207	2774	145	88	86	3300

DALY, disability-adjusted life year; GDP, gross domestic product; LMIC, low and middle-income country.

**Table 3:** Cost-saving interventions in LMICs (2008–2017). n (cost-saving ratios)=207; n (articles)=33

Targeted region*	n (ratios) (%)
Central Europe, Eastern Europe and Central Asia	5 (2%)
Latin America and Caribbean	24 (12%)
North Africa and Middle East	24 (12%)
South Asia	13 (6%)
Southeast Asia, East Asia and Oceania	58 (28%)
Sub-Saharan Africa	110 (53%)
**GBD classification (n=207**)	
Communicable maternal, neonatal and nutritional disorders (n=126; 61%) Frequency missing=3	
Diarrhoea, lower respiratory infections, meningitis and other common infectious diseases	18 (15%)
HIV/AIDS and tuberculosis	57 (46%)
Maternal disorders	3 (2%)
Neglected tropical diseases and malaria	23 (19%)
Other communicable, maternal, neonatal and nutritional disorders	22 (18%)
Total	123
Non-communicable diseases (n=81; 39%) Frequency missing=1	
Mental and behavioural disorders	23 (28%)
Cardiovascular and circulatory diseases	4 (5%)
Diabetes, urogenital, blood and endocrine diseases	6 (8%)
Neoplasms	47 (59%)
Total	80
**Inclusion of implementation costs (n=207**)	120 (58%)
Specific components included	
Infrastructure	46 (22%)
Administrative	83 (40%)
Salary	72 (35%)
Other	31 (15%)

*Ratios can encompass more than one region (16 ratios target multiple countries). Therefore, the counts will add to greater than 100%.

GBD, Global Burden of Disease; LMIC, low and middle-income country.

In addition, while 58% of cost-saving ratios (120/207) included costs associated directly with the implementation of the intervention, 22% accounted for infrastructure, 40% administrative and 35% salary support (table 3).

### Inclusion of cost-saving interventions in countries’ policy planning

For the 10 countries we investigated in sub-Saharan Africa, we identified 17 studies detailing 45 interventions that were cost saving. When we accounted for 12 cost-saving interventions that applied to more than one country, there were 137 sets of savings and health gains across countries. From our sample of 45 unique interventions, 36% (16/45) included costs associated directly with the implementation of the intervention; 16% accounted for infrastructure costs, 31% administrative and 22% salary support.

We found that 58% (79/137) of cost-saving interventions correspond with priorities identified in country plans (table 4). Concordance, however, varied across country and disease area. Alignment ranged from 95% (21/22 prioritised cost-saving ratios) in South Africa to 17% (2/12 prioritised cost-saving ratios) in Cameroon (table 4).

**Table 4:** Cost-saving interventions included in LMIC health prioritisation plans (10 sampled countries in sub-Saharan Africa, total of 137 sets of savings)

Country	n (ratios) (%)	✓	–	X
Low-income economies*				
Liberia	12 (8%)	3 (25%)	9 (75%)	0 (0%)
Malawi	16 (11%)	13 (81%)	0 (0%)	3 (19%)
Sierra Leone	12 (8%)	11 (92%)	0 (0%)	1 (8%)
Lower middle-income economies				
Cameroon	12 (9%)	2 (17%)	0 (0%)	10 (83%)
Lesotho	12 (8%)	4 (33%)	8 (67%)	0 (0%)
Nigeria	13 (9%)	11 (85%)	2 (15%)	0 (0%)
Zambia	14 (10%)	6 (43%)	8 (57%)	0 (0%)
Zimbabwe	14 (10%)	6 (43%)	8 (57%)	0 (0%)
Upper middle-income economies				
South Africa	22 (15%)	21 (95%)	1 (5%)	0 (0%)
Swaziland	10 (7%)	2 (20%)	0 (0%)	8 (80%)
Total	137	79 (58%)	36 (26%)	22 (16%)

✓Available policy documentation matches cost-saving intervention area.

– Cost-saving intervention area not explicitly specified.

X Cost-saving intervention area not mentioned in available policy documentation.

*Defined by the World Bank (https://datahelpdesk.worldbank.org/knowledgebase/articles/906519).

LMIC, low and middle-income country.

The following initiatives were considered cost saving in each of the 10 countries in our sample: human papillomavirus (HPV) vaccination, antenatal syphilis screening and HIV prevention and treatment initiatives (such as expanded treatment, circumcision, HIV self-testing and initiatives for pregnant women to prevent child transmission) (online supplementary appendix table 2). HPV vaccination was a noted priority in 70% (7/10) of the national health prioritisation plans, while 40% (4/10) of countries explicitly included prenatal serological screening for syphilis. HIV prevention and treatment strategies were stated priorities in most country health plans; although Cameroon’s plans did not explicitly outline efforts for male circumcision (online supplementary appendix table 2).

Moreover, a global programme to eliminate lymphatic filariasis was considered cost saving in half of our sampled countries (Malawi, Nigeria, Sierra Leone, Cameroon and Liberia), two (40%) of which principally outlined efforts for lymphatic filariasis in their health policy priority plans. Online supplementary appendix table 2 details country-specific cost-saving interventions and their alignment with country national health plans.

## Discussion

Most countries prioritised cost-saving interventions for HPV and HIV prevention and treatment. Country prioritisation plans, however, either omitted or were ambiguous with regard to other cost-saving interventions, such as antenatal syphilis screening and efforts to address specific neglected tropical diseases. Given the burden of congenital syphilis in sub-Saharan Africa, the WHO recommends screening all women for syphilis at her first antenatal care visit, regardless of regional prevalence.^25^ Global efforts to eliminate neglected tropical diseases also remain a key health priority area for governments, with projected savings in the billions.^26 27^

Data availability and capacity constraints could have hindered comprehensive policy planning. Other factors such as donor funding, implementation limits, equity issues or political realities^28^ may also play a larger role in decision-making than economic evidence from cost/DALY studies. While cost-effectiveness information can help prioritise ‘high-value’ interventions, it might not capture short-term operational costs that could reduce or eliminate projected savings.^6 18^ Indeed, only 36% of the sampled interventions included costs associated directly with the implementation of a service. Moreover, others have suggested that rather than prioritising resources based on efficiency, governments should instead target health conditions imposing the greatest population burden.^18 19 29^ That strategy, however, can omit particularly vulnerable groups such as those affected by neglected disease areas.^26 30 31^

General limitations of this study include reliance on publicly available health policy documentation, which may be incomplete. For example, we collected some information regarding donor–government partnership, as donor plans often stated a collaborative partnership with applicable country ministries. However, our analysis may not capture a full range of multistakeholder partnership not listed in the policy documentation, and the level of the partnership can vary across countries. Moreover, our study relies on health policy goals rather than on direct measures of implementation. For instance, while Sierra Leone’s basic package of essential health services (2015–2020) outlined goals for syphilis testing at antenatal care clinics, a recent study revealed that syphilis detection and treatment services were available in fewer than 5% of antenatal care facilities in Sierra Leone in 2015.^32 33^ Further, our study takes a conservative approach to valuing services by identifying interventions deemed ‘cost-saving’, that is, interventions that are projected to both improve population health and reduce costs. Other initiatives have outlined interventions that are cost-effective or highly cost-effective based on predefined thresholds.^5^

Notwithstanding general study limits, these findings and corresponding appendices can be used by governments and other localised efforts to identify potential opportunities to reallocate resources to take advantage of highly efficient health interventions that they may not have been previously targeting.

## Conclusion

Reflecting on the importance of using economic evidence in country priority setting, our paper assessed whether LMICs have included ‘cost-saving’ health interventions in national health priority plans. Our findings indicate substantial variation across country and disease area in how a sample of countries in sub-Saharan Africa incorporate economic evidence into national health priority plans. Countries may not place substantial emphasis on health economic findings because CEAs often omit important cost elements. For example, we found that fewer than half of the interventions in our sample included costs associated directly with the implementation of the intervention. Operational costs and other contextual factors may play a larger role than economic evidence in decision-making. To make health economic data more salient, authors of cost-effectiveness studies must do more to reflect other factors that could limit healthcare delivery. Future research may also address why some countries are integrating economic evidence into their policy planning compared with others. Donors and other competing factors could also be influential factors explaining these differences.
