# Supplementary material for: Are low and middle-income countries prioritising high-value healthcare interventions?
Source: BMJ Glob Health. 2020 Feb 4;5(2):e001850. doi: 10.1136/bmjgh-2019-001850 (PMC7042606; doi:10.1136/bmjgh-2019-001850)
Supplement: Supplementary data [file bmjgh-2019-001850supp001.pdf]

APPENDIX

Tables 1 and 2 below are extracted from the Tufts Medical Center Global Health Cost-Effectiveness Analysis (GHCEA) Registry, a repository of English-language cost-per-DALY studies indexed in PubMed that is sponsored by the Bill and Melinda Gates Foundation (ghcearegistry.org). The methodology of the GHCEA registry closely follows that of the CEA Registry (cearegistry.org) (a database of 8,056 cost-utility publications/analyses published from 1976 to 2018). A team of researchers systematically searches PubMed for articles with titles or abstracts containing either “disability-adjusted” or “DALY.” Every study that is part of the registry undergoes a formal review with a quality assurance protocol. Two reviewers trained in decision science and cost-effectiveness methodology independently screen each publication and extract data using a set of standardized forms. Data on methodology, interventions, and health outcomes are collected, and the two reviewers then meet for a consensus audit to resolve any discrepancies. The search protocols and inclusion criteria for the CEA Registry have been detailed elsewhere (1,2).

Appendix, Table 1: Cost-Saving Interventions in LMICs (2008-2017)

N	GBD classification	Intervention	Comparator	Country/ Region	Target Population	Perspective	Time horizon
**1**	HIV/AIDS	HIV-ab testing screening of blood donations	None	Ghana	All persons > 2 years of age	Limited Societal	Lifetime
**2**	HIV/AIDS	Efavirenz-based regimens	Nevirapine-based regimens	Thailand	All persons aged 50	Healthcare Payer	Lifetime
**3**	HIV/AIDS	Efavirenz-based regimens	Nevirapine-based regimens	Thailand	All persons aged 60	Healthcare Payer	Lifetime
**4**	HIV/AIDS	Efavirenz-based regimens	Nevirapine-based regimens	Thailand	All persons aged 30	Healthcare Payer	Lifetime
**5**	HIV/AIDS	Efavirenz-based regimens	Nevirapine-based regimens	Thailand	All persons aged 40	Healthcare Payer	Lifetime
**6**	HIV/AIDS	Efavirenz-based regimens	Nevirapine-based regimens	Thailand	All persons aged 15-65	Healthcare Payer	Lifetime
**7**	HIV/AIDS	Efavirenz-based regimens	Nevirapine-based regimens	Thailand	All persons aged 15-65	Healthcare Payer	Lifetime
**8**	HIV/AIDS	Efavirenz-based regimens	Nevirapine-based regimens	Thailand	All persons aged 15-65	Healthcare Payer	Lifetime
**9**	HIV/AIDS	Efavirenz-based regimens	Nevirapine-based regimens	Thailand	All persons aged 15-65	Healthcare Payer	Lifetime
**10**	HIV/AIDS	Efavirenz-based regimens	Nevirapine-based regimens	Thailand	All persons aged 15-65	Healthcare Payer	Lifetime

**11**	Cervical cancer	Human papilloma virus 16/18 vaccination; vaccine cost of I$25	Standard/Usual Care	Barbados	Females aged 9	Limited Societal	Lifetime
**12**	Tuberculosis	Rapid drug susceptibility testing (DST) using direct amplification and reverse hybridization of the rpoB gene (INNOLiPA; Innogenetics) for the detection of Rif resistance	rapid drug susceptibility testing (DST) using indirect proportion method on Lo¨wenstein-Jensen medium (IDLJ) for the detection of Rif resistance	Peru	All persons, target age unknown	Healthcare Payer	Lifetime
**13**	Tuberculosis	Rapid drug susceptibility testing (DST) using direct phage-replication assay (FASTPlaque-Response; Biotech) for the detection of Rif resistance	rapid drug susceptibility testing (DST) using indirect proportion method on Lo¨wenstein-Jensen medium (IDLJ) for the detection of Rif resistance	Peru	All persons, target age unknown	Healthcare Payer	Lifetime
**14**	Tuberculosis	Rapid drug susceptibility testing (DST) using indirect proportion method on Lo¨wenstein-Jensen medium (IDLJ) for the detection of rif resistance	None	Peru	All persons, target age unknown	Healthcare Payer	Lifetime
**15**	Tuberculosis	Rapid drug susceptibility testing (DST) using the direct amplification and reverse hybridization of the rpoB gene (INNOLiPA; Innogenetics) for detection of multi drug resistance	None	Peru	All persons, target age unknown	Healthcare Payer	Lifetime
**16**	Tuberculosis	Rapid drug susceptibility testing (DST) using the direct phage-replication assay (FASTPlaque-Response; Biotech) for detection of multi drug resistance	Rapid drug susceptibility testing (DST) using the indirect proportion method on Lo¨wenstein-Jensen medium (IDLJ) for detection of multi drug resistance	Peru	All persons, target age unknown	Healthcare Payer	Lifetime
**17**	Tuberculosis	Rapid drug susceptibility testing (DST) using the direct proportion method on Lo¨wenstein-Jensen medium (DLJ) for detection of multi drug resistance	Rapid drug susceptibility testing (DST) using the indirect proportion method on Lo¨wenstein-Jensen medium (IDLJ) for detection of multi drug resistance	Peru	All persons, target age unknown	Healthcare Payer	Lifetime

**18**	Tuberculosis	Rapid drug susceptibility testing (DST) using the direct proportion method on Lo¨wenstein-Jensen medium (DLJ) for the detection of Rif resistance	rapid drug susceptibility testing (DST) using indirect proportion method on Lo¨wenstein-Jensen medium (IDLJ) for the detection of Rif resistance	Peru	All persons, target age unknown	Healthcare Payer	Lifetime
**19**	Tuberculosis	Rapid drug susceptibility testing (DST) using the indirect colorimetric minimum inhibitory concentration assay (MTT; ICN Biomedicals) for the detection of Rif resistance	rapid drug susceptibility testing (DST) using indirect proportion method on Lo¨wenstein-Jensen medium (IDLJ) for the detection of Rif resistance	Peru	All persons, target age unknown	Healthcare Payer	Lifetime
**20**	Tuberculosis	Rapid drug susceptibility testing (DST) using the indirect colorimetric minimum inhibitory concentration assay (MTTassay; ICN Biomedicals) for detection of multi drug resistance	None	Peru	All persons, target age unknown	Healthcare Payer	Lifetime
**21**	Tuberculosis	Rapid drug susceptibility testing (DST) using the indirect proportion method on Lo¨wenstein-Jensen medium (IDLJ) for detection of multi drug resistance	None	Peru	All persons, target age unknown	Healthcare Payer	Lifetime
**22**	Sexually transmitted diseases excluding HIV	HPV 16, 18 vaccination	None	Comoros	Females aged 9	Healthcare Payer	Lifetime
**23**	Sexually transmitted diseases excluding HIV	HPV 16, 18 vaccination	None	Djibouti	Females aged 9	Healthcare Payer	Lifetime
**24**	Sexually transmitted diseases excluding HIV	HPV 16, 18 vaccination	None	Eritrea	Females aged 9	Healthcare Payer	Lifetime
**25**	Sexually transmitted diseases excluding HIV	HPV 16, 18 vaccination	None	Guinea	Females aged 9	Healthcare Payer	Lifetime

**26**	Sexually transmitted diseases excluding HIV	HPV 16, 18 vaccination	None	Guyana	Females aged 9	Healthcare Payer	Lifetime
**27**	Sexually transmitted diseases excluding HIV	HPV 16, 18 vaccination	None	Lesotho	Females aged 9	Healthcare Payer	Lifetime
**28**	Sexually transmitted diseases excluding HIV	HPV 16, 18 vaccination	None	Nicaragua	Females aged 9	Healthcare Payer	Lifetime
**29**	Sexually transmitted diseases excluding HIV	HPV 16, 18 vaccination	None	Rwanda	Females aged 9	Healthcare Payer	Lifetime
**30**	Sexually transmitted diseases excluding HIV	HPV 16, 18 vaccination	None	Uganda	Females aged 9	Healthcare Payer	Lifetime
**31**	Sexually transmitted diseases excluding HIV	HPV 16, 18 vaccination	None	Zimbabwe	Females aged 9	Healthcare Payer	Lifetime
**32**	HIV/AIDS	Pregnant women counseled, tested, and given highly active antiretroviral therapy for prevention of mother to child transmission of HIV	None	Malawi	Pregnant women & infants	Limited Societal	Lifetime
**33**	Other communicable, maternal, neonatal, and nutritional disorders	Global Polio Eradication Initiative then no routine vaccination	Standard/Usual Care-Routine vaccination	Multiple Countries	All persons, target age unknown	Healthcare Payer	47 years
**34**	Other communicable, maternal, neonatal, and	Global Polio Eradication Initiative then universal IPV	Standard/Usual Care-Routine vaccination	Multiple Countries	All persons, target age unknown	Healthcare Payer	47 years

	nutritional disorders						
**35**	HIV/AIDS	Hematocrit test conducted at baseline (asymptomatic)	None	Haiti	All persons 16 years of age & older	Healthcare Payer	Lifetime
**36**	Diarrheal diseases	Quality Intervention (QI) to improve pediatric hospital care, specifically for diarrhea and pneumonia	Standard/Usual Care	Nicaragua	Infants < 5	Healthcare Payer	2 years
**37**	Other maternal disorders	Gestational diabetes mellitus (GDM) screen and life style change	None	India	Pregnant women	Healthcare Payer	MISSING
**38**	Sexually transmitted diseases excluding HIV	New dual treponemal/nontreponemal point-of-care test (Dual-POC) for prenatal serological screening	None	Multiple Countries	Pregnant women	Healthcare Sector	Lifetime
**39**	Sexually transmitted diseases excluding HIV	New dual treponemal/nontreponemal point-of-care test (Dual-POC) for prenatal serological screening	None	Multiple Countries	Infants < 5	Healthcare Sector	Lifetime
**40**	Sexually transmitted diseases excluding HIV	Point-of-care treponemal immunochromatographic strip (ICS) test for prenatal serological screening	None	Multiple Countries	Pregnant women	Healthcare Sector	Lifetime
**41**	Sexually transmitted diseases excluding HIV	Point-of-care treponemal immunochromatographic strip (ICS) test for prenatal serological screening	None	Multiple Countries	Infants < 5	Healthcare Sector	Lifetime
**42**	Sexually transmitted diseases excluding HIV	Rapid plasma reagin plus Treponema pallidum passive particule agglutination assay for prenatal serological screening	None	Multiple Countries	Pregnant women	Healthcare Sector	Lifetime
**43**	Sexually transmitted diseases excluding HIV	Rapid plasma reagin plus Treponema pallidum passive particule agglutination assay for prenatal serological screening	None	Multiple Countries	Infants < 5	Healthcare Sector	Lifetime
**44**	Sexually transmitted diseases excluding HIV	Rapid plasma regain (RPR) test for prenatal serological screening	None	Multiple Countries	Pregnant women	Healthcare Sector	Lifetime

**45**	Sexually transmitted diseases excluding HIV	Rapid plasma regain (RPR) test for prenatal serological screening	None	Multiple Countries	Infants < 5	Healthcare Sector	Lifetime
**46**	Malaria	Parental artesunate	Standard/Usual Care-Quinine dihyrocholride	Tanzania	All persons between the ages 0-18	Healthcare Payer	Lifetime
**47**	Diarrheal diseases	Human papillomavirus (HPV) vaccine	None	Comoros	Infants < 5	Healthcare Payer	Lifetime
**48**	Diarrheal diseases	Human papillomavirus (HPV) vaccine	None	Djibouti	Infants < 5	Healthcare Payer	Lifetime
**49**	Diarrheal diseases	Human papillomavirus (HPV) vaccine	None	Eritrea	Infants < 5	Healthcare Payer	Lifetime
**50**	Diarrheal diseases	Human papillomavirus (HPV) vaccine	None	Guinea	Infants < 5	Healthcare Payer	Lifetime
**51**	Diarrheal diseases	Human papillomavirus (HPV) vaccine	None	Guyana	Infants < 5	Healthcare Payer	Lifetime
**52**	Diarrheal diseases	Human papillomavirus (HPV) vaccine	None	Lesotho	Infants < 5	Healthcare Payer	Lifetime
**53**	Diarrheal diseases	Human papillomavirus (HPV) vaccine	None	Mauritania	Infants < 5	Healthcare Payer	Lifetime
**54**	Diarrheal diseases	Human papillomavirus (HPV) vaccine	None	Nicaragua	Infants < 5	Healthcare Payer	Lifetime
**55**	Diarrheal diseases	Human papillomavirus (HPV) vaccine	None	Rwanda	Infants < 5	Healthcare Payer	Lifetime
**56**	Diarrheal diseases	Human papillomavirus (HPV) vaccine	None	Uganda	Infants < 5	Healthcare Payer	Lifetime
**57**	Diarrheal diseases	Human papillomavirus (HPV) vaccine	None	Zimbabwe	Infants < 5	Healthcare Payer	Lifetime
**58**	Diarrheal diseases	Rotavirus vaccine	None	Angola	Infants < 5	Healthcare Payer	Lifetime
**59**	Schizophrenia	Clozapine to patients with highest sevirity (risperidone to remaing) plus family intervention	None	Thailand	All adult persons, age not specified	Limited Societal	Lifetime
**60**	Schizophrenia	Risperidone	None	Thailand	All adult persons, age not specified	Limited Societal	Lifetime
**61**	Schizophrenia	Risperidone plus family intervention consisting of weekly sessions with psychiatric nurse	None	Thailand	All adult persons, age not specified	Limited Societal	Lifetime

**62**	Schizophrenia	Typical antipsychotics	None	Thailand	All adult persons, age not specified	Limited Societal	Lifetime
**63**	Drug use disorders	Excise tax increase	None	Vietnam	All persons, target age unknown	Healthcare Payer	10 years
**64**	Drug use disorders	Graphic warning labels on cigarette packs	None	Vietnam	All persons, target age unknown	Healthcare Payer	10 years
**65**	Drug use disorders	Mass media campaign	None	Vietnam	All persons, target age unknown	Healthcare Payer	10 years
**66**	Drug use disorders	Smoking ban	None	Vietnam	All persons, target age unknown	Healthcare Payer	10 years
**67**	HIV/AIDS	Circumcision for prevention of heterosexual acquisition of HIV	None	Multiple Countries	Adult males, age not specified	Healthcare Payer	Lifetime
**68**	Diarrheal diseases	2-dose rotavirus vaccination program	None	China	Infants < 5	Limited Societal	Lifetime
**69**	Other endocrine, nutritional, blood, and immune disorders	Congenital hypothyroidism screening with thyroid-stimulating hormone (TSH), cut-off point= 10mU/l	None	Iran, Islamic Rep.	Infants 3-5 days old	Limited Societal	82 years
**70**	Missing	Universal access to modern contraceptives	Standard/Usual Care- current contraceptive program	Uganda	Females aged 15	Limited Societal	Lifetime
**71**	HIV/AIDS	Expanding antiretroviral treatment (ART) to CD4 count <350 + current prevention	Standard/Usual Care- Expanding antiretroviral treatment (ART) to CD4 count <200 + current prevention	South Africa	All persons aged 15-49	Healthcare Payer	40 years
**72**	HIV/AIDS	Expanding antiretroviral treatment (ART) to CD4 count <350 + enhanced prevention	Standard/Usual Care- Expanding antiretroviral treatment (ART) to CD4 count <200 + enhanced prevention	South Africa	All persons aged 15-49	Healthcare Payer	40 years
**73**	HIV/AIDS	Expanding antiretroviral treatment (ART) to CD4 count <500 + current prevention	Expanding antiretroviral treatment (ART) to CD4 count <350 + current prevention	South Africa	All persons aged 15-49	Healthcare Payer	40 years

**74**	HIV/AIDS	Expanding antiretroviral treatment (ART) to CD4 count <500 + enhanced prevention	Expanding antiretroviral treatment (ART) to CD4 count <350 + enhanced prevention	South Africa	All persons aged 15-49	Healthcare Payer	40 years
**75**	HIV/AIDS	Expanding antiretroviral treatment (ART) to all CD4 levels + current prevention	Expanding antiretroviral treatment (ART) to CD4 count <500 + current prevention	South Africa	All persons aged 15-49	Healthcare Payer	40 years
**76**	HIV/AIDS	Expanding antiretroviral treatment (ART) to all CD4 levels + current prevention	Standard/Usual Care-Expanding antiretroviral treatment (ART) to CD4 count <200 + current prevention	South Africa	All persons aged 15-49	Healthcare Payer	40 years
**77**	HIV/AIDS	Expanding antiretroviral treatment (ART) to all CD4 levels + enhanced prevention	Expanding antiretroviral treatment (ART) to CD4 count <500 + enhanced prevention	South Africa	All persons aged 15-49	Healthcare Payer	40 years
**78**	HIV/AIDS	Expanding antiretroviral treatment (ART) to all CD4 levels + enhanced prevention	Standard/Usual Care-Expanding antiretroviral treatment (ART) to CD4 count <200 + enhanced prevention	South Africa	All persons aged 15-49	Healthcare Payer	40 years
**79**	HIV/AIDS	Integrated HIV testing, malaria, and diarrhea prevention campaign	None	Kenya	All persons, aged unknown	Healthcare Payer	Lifetime
**80**	Tuberculosis	Patients' drug intake is supervised daily for 6 months by a family member (family-member DOT) at the patients' home.	Patients take their daily medication without supervision for 6 months.	Thailand	All persons aged 15+	Limited Societal	Lifetime
**81**	Tuberculosis	Patients' drug intake is supervised daily for 6 months by a village health volunteer	Patients take their daily medication without supervision for 6 months.	Thailand	All persons aged 15+	Limited Societal	Lifetime
**82**	Cervical cancer	Human papillomavirus (HPV) 16/18 vaccination	None	Algeria	Females up to 12 years	Limited Societal	Lifetime
**83**	Cervical cancer	Human papillomavirus (HPV) 16/18 vaccination	None	Bahrain	Females up to 12 years	Limited Societal	Lifetime
**84**	Cervical cancer	Human papillomavirus (HPV) 16/18 vaccination	None	Kuwait	Females up to 12 years	Limited Societal	Lifetime
**85**	Cervical cancer	Human papillomavirus (HPV) 16/18 vaccination	None	Morocco	Females up to 12 years	Limited Societal	Lifetime
**86**	Cervical cancer	Human papillomavirus (HPV) 16/18 vaccination	None	Oman	Females up to 12 years	Limited Societal	Lifetime
**87**	Cervical cancer	Human papillomavirus (HPV) 16/18 vaccination	None	Pakistan	Females up to 12 years	Limited Societal	Lifetime

**88**	Cervical cancer	Human papillomavirus (HPV) 16/18 vaccination	None	Qatar	Females up to 12 years	Limited Societal	Lifetime
**89**	Cervical cancer	Human papillomavirus (HPV) 16/18 vaccination	None	United Arab Emirates	Females up to 12 years	Limited Societal	Lifetime
**90**	Cervical cancer	HPV16/18 vaccination	None	Angola	Females up to 12 years	Limited Societal	Lifetime
**91**	Cervical cancer	HPV16/18 vaccination	None	Benin	Females up to 12 years	Limited Societal	Lifetime
**92**	Cervical cancer	HPV16/18 vaccination	None	Botswana	Females up to 12 years	Limited Societal	Lifetime
**93**	Cervical cancer	HPV16/18 vaccination	None	Burkina Faso	Females up to 12 years	Limited Societal	Lifetime
**94**	Cervical cancer	HPV16/18 vaccination	None	Burundi	Females up to 12 years	Limited Societal	Lifetime
**95**	Cervical cancer	HPV16/18 vaccination	None	Cabo Verde	Females up to 12 years	Limited Societal	Lifetime
**96**	Cervical cancer	HPV16/18 vaccination	None	Cameroon	Females up to 12 years	Limited Societal	Lifetime
**97**	Cervical cancer	HPV16/18 vaccination	None	Comoros	Females up to 12 years	Limited Societal	Lifetime
**98**	Cervical cancer	HPV16/18 vaccination	None	Congo, Rep.	Females up to 12 years	Limited Societal	Lifetime
**99**	Cervical cancer	HPV16/18 vaccination	None	Cote d'Ivoire	Females up to 12 years	Limited Societal	Lifetime
**100**	Cervical cancer	HPV16/18 vaccination	None	Equatorial Guinea	Females up to 12 years	Limited Societal	Lifetime
**101**	Cervical cancer	HPV16/18 vaccination	None	Gabon	Females up to 12 years	Limited Societal	Lifetime
**102**	Cervical cancer	HPV16/18 vaccination	None	Gambia, The	Females up to 12 years	Limited Societal	Lifetime
**103**	Cervical cancer	HPV16/18 vaccination	None	Ghana	Females up to 12 years	Limited Societal	Lifetime
**104**	Cervical cancer	HPV16/18 vaccination	None	Guinea	Females up to 12 years	Limited Societal	Lifetime
**105**	Cervical cancer	HPV16/18 vaccination	None	Guinea-Bissau	Females up to 12 years	Limited Societal	Lifetime
**106**	Cervical cancer	HPV16/18 vaccination	None	Kenya	Females up to 12 years	Limited Societal	Lifetime
**107**	Cervical cancer	HPV16/18 vaccination	None	Liberia	Females up to 12 years	Limited Societal	Lifetime

**108**	Cervical cancer	HPV16/18 vaccination	None	Madagascar	Females up to 12 years	Limited Societal	Lifetime
**109**	Cervical cancer	HPV16/18 vaccination	None	Malawi	Females up to 12 years	Limited Societal	Lifetime
**110**	Cervical cancer	HPV16/18 vaccination	None	Mauritania	Females up to 12 years	Limited Societal	Lifetime
**111**	Cervical cancer	HPV16/18 vaccination	None	Mauritius	Females up to 12 years	Limited Societal	Lifetime
**112**	Cervical cancer	HPV16/18 vaccination	None	Mozambique	Females up to 12 years	Limited Societal	Lifetime
**113**	Cervical cancer	HPV16/18 vaccination	None	Namibia	Females up to 12 years	Limited Societal	Lifetime
**114**	Cervical cancer	HPV16/18 vaccination	None	Nigeria	Females up to 12 years	Limited Societal	Lifetime
**115**	Cervical cancer	HPV16/18 vaccination	None	Rwanda	Females up to 12 years	Limited Societal	Lifetime
**116**	Cervical cancer	HPV16/18 vaccination	None	Sao Tome and Principe	Females up to 12 years	Limited Societal	Lifetime
**117**	Cervical cancer	HPV16/18 vaccination	None	Senegal	Females up to 12 years	Limited Societal	Lifetime
**118**	Cervical cancer	HPV16/18 vaccination	None	Seychelles	Females up to 12 years	Limited Societal	Lifetime
**119**	Cervical cancer	HPV16/18 vaccination	None	Sierra Leone	Females up to 12 years	Limited Societal	Lifetime
**120**	Cervical cancer	HPV16/18 vaccination	None	South Africa	Females up to 12 years	Limited Societal	Lifetime
**121**	Cervical cancer	HPV16/18 vaccination	None	Swaziland	Females up to 12 years	Limited Societal	Lifetime
**122**	Cervical cancer	HPV16/18 vaccination	None	Tanzania	Females up to 12 years	Limited Societal	Lifetime
**123**	Cervical cancer	HPV16/18 vaccination	None	Togo	Females up to 12 years	Limited Societal	Lifetime
**124**	Cervical cancer	HPV16/18 vaccination	None	Uganda	Females up to 12 years	Limited Societal	Lifetime
**125**	Cervical cancer	HPV16/18 vaccination	None	Zambia	Females up to 12 years	Limited Societal	Lifetime
**126**	Cervical cancer	HPV16/18 vaccination	None	Zimbabwe	Females up to 12 years	Limited Societal	Lifetime

**127**	Lower respiratory infections	Haemophilus influenzae Type b (Hib) vaccination	None	Multiple Countries	Infants < 5	Limited Societal	Lifetime
**128**	Maternal haemorrhage	Non-pneumatic anti-shock garment (NASG)	NASG if mean arterial pressure (MAP) <60 mmHg (severe shock)	Egypt, Arab Rep.	Pregnant women	Healthcare Payer	10 years
**129**	Maternal haemorrhage	Non-pneumatic anti-shock garment (NASG) if mean arterial pressure (MAP) <60 mmHg	None	Egypt, Arab Rep.	Pregnant women	Healthcare Payer	10 years
**130**	Alcohol use disorders	Mass media campaign against drunk driving	None	Thailand	All persons, age not specified	Healthcare Sector	Lifetime
**131**	Alcohol use disorders	Mass media campaign against drunk driving and random roadside breath testing	None	Thailand	All persons, age not specified	Healthcare Sector	Lifetime
**132**	Alcohol use disorders	Mass media campaign against drunk driving and selective roadside breath testing	None	Thailand	All persons, age not specified	Healthcare Sector	Lifetime
**133**	Alcohol use disorders	Random breath testing for Driving under the influence of alcohol	None	Thailand	All persons, age not specified	Healthcare Sector	Lifetime
**134**	Alcohol use disorders	Selective breath testing for Driving under the influence of alcohol	None	Thailand	All persons, age not specified	Healthcare Sector	Lifetime
**135**	HIV/AIDS	Expanded eligibility for HIV + antiretroviral therapy treatment to all HIV + patients and expanded access in routine HIV testing and linkage to care such that 80% of HIV + infected persons will receive care when eligible for treatment	Implementation of program to expand eligibility for HIV + Antiretroviral therapy treatment to patients with CD4<500 cells/ml and to expand access in routine HIV testing and linkage to care such that 80% of HIV + infected persons will receive care when they are eligible for treatment	Zambia	All persons equal or greater than 15 years of age	Healthcare Sector	20 years
**136**	HIV/AIDS	Expanded eligibility for HIV + antiretroviral therapy treatment to all HIV + patients and expanded access in routine HIV testing and linkage to care such	Implementation of program to expanded access in routine HIV testing and linkage to care such that 80% of HIV + infected persons will receive	Zambia	All persons equal or greater than 15 years of age	Healthcare Sector	20 years

		that 80% of HIV + infected persons will receive care when eligible for treatment	care when they are eligible for treatment				
**137**	Malaria	RTS,S vaccine to prevent malaria	None	Malawi	Infants, 5-12 months old	Limited Societal	Lifetime
**138**	Malaria	RTS,S vaccine to prevent malaria	Wide-spread provision of long-lasting insecticide-treated nets (LLINs) to newborns	Malawi	Infants, 5-12 months old	Limited Societal	Lifetime
**139**	Malaria	Wide-spread provision of long-lasting insecticide-treated nets (LLINs) to newborns	None	Malawi	Newborns	Limited Societal	Lifetime
**140**	Tuberculosis	Strengthened standardised retreatment regimen (second-line drugs)	Standard/Usual Care-Standard WHO recommended treatment strategy	Ecuador	All persons, age not specified	Limited Societal	10 years
**141**	Cardiomyopathy and myocarditis	Polypill (combining aspirin, beta blockers, angiotensin-converting enzyme inhibitors and statins) for prevention of acute myocardial infarction	Aspirin + beta blockers + angiotensin-converting enzyme inhibitors (ACEI) + statin	India	All adult persons, age not specified	Healthcare Sector	Lifetime
**142**	Malaria	Dihydroartemisinin piperaquine for first line treatment of plasmodium falciparum malaria (childhood malaria)	Standard/Usual Care-Artemether-Lumefantrine for first line treatment	Multiple Countries	Infants, 6-59 months	Healthcare Sector	1 year
**143**	Rheumatic heart disease	Acute rheumatic fever and rheumatic heart disease control program: education, training, register development, and improved adherence program	None	Cuba	All persons, aged 5-24	Healthcare Payer	Lifetime
**144**	HIV/AIDS	HIV self-testing	None	Zimbabwe	All persons, aged 15-65	Healthcare Payer	20 years
**145**	Schizophrenia	Olanzapine	None	Vietnam	All persons, aged 15+	Healthcare Payer	Lifetime
**146**	Schizophrenia	Risperidone	None	Vietnam	All persons, aged 15+	Healthcare Payer	Lifetime
**147**	Schizophrenia	Risperidone + clozapine	None	Vietnam	All persons, aged 15+	Healthcare Payer	Lifetime
**148**	Schizophrenia	Risperidone + olanzapine	None	Vietnam	All persons, aged 15+	Healthcare Payer	Lifetime

**149**	Schizophrenia	Risperidone plus family intervention	None	Vietnam	All persons, aged 15+	Healthcare Payer	Lifetime
**150**	Schizophrenia	Typical antipsychotic drugs	None	Vietnam	All persons, aged 15+	Healthcare Payer	Lifetime
**151**	Schizophrenia	Typical antipsychotic drugs + clozapine	None	Vietnam	All persons, aged 15+	Healthcare Payer	Lifetime
**152**	Schizophrenia	Typical antipsychotic drugs + olanzapine	None	Vietnam	All persons, aged 15+	Healthcare Payer	Lifetime
**153**	Schizophrenia	Typical antipsychotic drugs + risperidone	None	Vietnam	All persons, aged 15+	Healthcare Payer	Lifetime
**154**	Schizophrenia	Typical antipsychotic drugs plus family intervention	None	Vietnam	All persons, aged 15+	Healthcare Payer	Lifetime
**155**	Iron-deficiency anemia	Price subsidy accounting for 50% of cost fortified packaged infant cereals; lowest income quintile families eligible	None	India	Infants 6-23 months	Limited Societal	Lifetime
**156**	Neglected tropical diseases and malaria	Annual mass drug administration (MDA) of the Hookworm vaccine: 80% efficacy, 10 year duration of protection, 97% coverage, no booster	None	Brazil	Infants 5-14 months	Limited Societal	Lifetime
**157**	Neglected tropical diseases and malaria	Annuals mass drug administration (MDA); 56.2% coverage, $0.21 per child treated, 2010 GBD disability rate used	None	Brazil	Infants 5-14 months	Limited Societal	Lifetime
**158**	Breast cancer	Breast Cancer Screening using Mammography	Standard/Usual Care	Iran, Islamic Rep.	Females aged 35-69	Healthcare Payer	Lifetime
**159**	Diabetes mellitus	Benefit-based tailored treatment	Target-to-treat	China	All persons aged 20-79	Healthcare Sector	10 years
**160**	Diabetes mellitus	Benefit-based tailored treatment	Target-to-treat	Ghana	All persons aged 20-79	Healthcare Sector	10 years
**161**	Diabetes mellitus	Benefit-based tailored treatment	Target-to-treat	India	All persons aged 20-79	Healthcare Sector	10 years
**162**	Diabetes mellitus	Benefit-based tailored treatment	Target-to-treat	Mexico	All persons aged 20-79	Healthcare Sector	10 years
**163**	Diabetes mellitus	Benefit-based tailored treatment	Target-to-treat	South Africa	All persons aged 20-79	Healthcare Sector	10 years

**164**	Tuberculosis	Combination (expand access to care; active case finding in the general population; introduce Xpert MTB/RIF for diagnosis; improve treatment quality)	Standard/Usual Care- base case levels	India	All persons, age not specified	Limited Societal	10 years
**165**	Tuberculosis	Expand access to tuberculosis treatment	NoneStandard/Usual Care- base case levels	India	All persons, age not specified	Limited Societal	10 years
**166**	Tuberculosis	Expand access to tuberculosis treatment	Standard/Usual Care- base case levels	China	All persons, age not specified	Limited Societal	10 years
**167**	Tuberculosis	Improve treatment for HIV and tuberculosis	Standard/Usual Care- base case levels	India	All persons, age not specified	Limited Societal	10 years
**168**	Tuberculosis	Improve tuberculosis treatment	Standard/Usual Care- base case levels	China	All persons, age not specified	Limited Societal	10 years
**169**	Ischemic heart disease	Legal limit on dietary trans fat	No EU action	Multiple Countries	All persons, age not specified	Limited Societal	Lifetime
**170**	Ischemic heart disease	Voluntary agreements on dietary trans fat limit	No EU action	Multiple Countries	All persons, age not specified	Limited Societal	Lifetime
**171**	HIV/AIDS	ART initiation with CD4 count > 500	Deferred ART initiation until decline in CD4 reaches < 350, development of AIDS, or development of other conditions necessitating ART initiation	India	All persons aged 19-40 years	Healthcare Payer	5 years
**172**	HIV/AIDS	ART initiation with CD4 count > 500	Deferred ART initiation until decline in CD4 reaches < 350, development of AIDS, or development of other conditions necessitating ART initiation	Nigeria	All persons aged 19-40 years	Healthcare Payer	5 years
**173**	HIV/AIDS	ART initiation with CD4 count > 500	Deferred ART initiation until decline in CD4 reaches < 350, development of AIDS, or development of other	South Africa	All persons aged 19-40 years	Healthcare Payer	5 years

			conditions necessitating ART initiation				
**174**	HIV/AIDS	ART initiation with CD4 count > 500	Deferred ART initiation until decline in CD4 reaches < 350, development of AIDS, or development of other conditions necessitating ART initiation	Uganda	All persons aged 19-40 years	Healthcare Payer	5 years
**175**	Lymphatic filariasis	Global Programme to Eliminate Lymphatic Filariasis (GPELF)	None	Multiple Countries	All adults at risk, age not specified	Limited Societal	Lifetime
**176**	Tuberculosis	Bedaquiline + background regimen	Standard/Usual Care	China	All adult persons, age not specified	Healthcare Payer	10 years
**177**	Tuberculosis	Bedaquiline + background regimen	Standard/Usual Care	Estonia	All adult persons, age not specified	Healthcare Payer	10 years
**178**	Tuberculosis	Bedaquiline + background regimen	Standard/Usual Care	India	All adult persons, age not specified	Healthcare Payer	10 years
**179**	Tuberculosis	Bedaquiline + background regimen	Standard/Usual Care	Peru	All adult persons, age not specified	Healthcare Payer	10 years
**180**	Tuberculosis	Bedaquiline + background regimen	Standard/Usual Care	Philippines	All adult persons, age not specified	Healthcare Payer	10 years
**181**	Tuberculosis	Bedaquiline + background regimen	Standard/Usual Care	Russian Federation	All adult persons, age not specified	Healthcare Payer	10 years
**182**	Tuberculosis	Bedaquiline + background regimen	Standard/Usual Care	South Africa	All adult persons, age not specified	Healthcare Payer	10 years
**183**	Lower respiratory infections	13-valent pnumococcal conjugate vaccine	None	Mongolia	Infants < 5	Limited Societal	Lifetime
**184**	Malaria	Cotrimoxazole (CTX)	2-IPT Low (intermittent preventive treatment with sulfadoxine pyrimethamine)	Mozambique	Adult females	Limited Societal	40 weeks

**185**	Tuberculosis	Bedaquiline for extensively drug-resistant tuberculosis and standard regimen for multi-drug resistant/rifampicin-resistant tuberculosis	Standard/Usual Care	South Africa	All adult persons, age not specified	Healthcare Payer	10 years
**186**	Communicable maternal, neonatal, and nutritional disorders	Enterovirus 71 vaccination at $2.5 per dose	None	China	Infants < 5	Limited Societal	5 years
**187**	Communicable maternal, neonatal, and nutritional disorders	Enterovirus 71 vaccination at $5 per dose	None	China	Infants < 5	Limited Societal	5 years
**188**	Communicable maternal, neonatal, and nutritional disorders	Enterovirus 71 vaccination at $5.2 per dose	None	China	Infants < 5	Limited Societal	5 years
**189**	Neglected tropical diseases and malaria	Indoor residual spraying 100%	None	Mozambique	General population, central region	Limited Societal	3 years
**190**	Neglected tropical diseases and malaria	Indoor residual spraying 100%	None	Mozambique	General population, southern region	Limited Societal	3 years
**191**	Neglected tropical diseases and malaria	Indoor residual spraying 80%	None	Mozambique	General population, southern region	Limited Societal	3 years
**192**	Neglected tropical diseases and malaria	Indoor residual spraying at 100%	None	Mozambique	General population, central region	Limited Societal	3 years
**193**	Neglected tropical diseases and malaria	Indoor residual spraying at 100%	None	Mozambique	General population, southern region	Limited Societal	3 years

**194**	Neglected tropical diseases and malaria	Long lasting insecticide nets at 100%	None	Mozambique	General population, central region	Limited Societal	3 years
**195**	Neglected tropical diseases and malaria	Long lasting insecticide nets at 100%	None	Mozambique	General population, central region	Limited Societal	3 years
**196**	Neglected tropical diseases and malaria	Long lasting insecticide nets at 100%	None	Mozambique	General population, northern region	Limited Societal	3 years
**197**	Neglected tropical diseases and malaria	Long lasting insecticide nets at 100%	None	Mozambique	General population, northern region	Limited Societal	3 years
**198**	Neglected tropical diseases and malaria	Long lasting insecticide nets at 100%	None	Mozambique	General population, southern region	Limited Societal	3 years
**199**	Neglected tropical diseases and malaria	Long lasting insecticide nets at 100%	None	Mozambique	General population, southern region	Limited Societal	3 years
**200**	Neglected tropical diseases and malaria	Long lasting insecticide nets at 80%	None	Mozambique	General population, central region	Limited Societal	3 years
**201**	Neglected tropical diseases and malaria	Long lasting insecticide nets at 80%	None	Mozambique	General population, northern region	Limited Societal	3 years
**202**	Neglected tropical diseases and malaria	Long lasting insecticide nets at 80%	None	Mozambique	General population, southern region	Limited Societal	3 years
**203**	Typhoid and paratyphoid fevers	Routine typhoid conjugate vaccine	None	India	Infants, 9 months	Healthcare Payer	10 years

**204**	Typhoid and paratyphoid fevers	Routine typhoid conjugate vaccine	None	Vietnam	Infants, 9 months	Healthcare Payer	10 years
**205**	Hepatitis	Directly-acting antiviral-based treatment	None	India	All persons, 35 years	Healthcare Payer	Lifetime
**206**	Tuberculosis	Light-emitting diode (LED) fluorescence microscopy using two sputum samples provided on the same day (FN-Spot-Spot)	Standard/Usual Care- Ziehl-Neelsen microscopy using three sputum samples provided within two days (ZN-Spot-Morning-Spot)	Ethiopia	All adult persons, age not specified	Healthcare Payer	10 years
**207**	Tuberculosis	Light-emitting diode (LED) fluorescence microscopy using two sputum samples provided within two days (FN-Spot-Morning)	Standard/Usual Care- Ziehl-Neelsen microscopy using three sputum samples provided within two days (ZN-Spot-Morning-Spot)	Ethiopia	All adult persons, age not specified	Healthcare Payer	10 years

Appendix, Table 2: Sampled countries/interventions (2008-2017)
Malawi, Zimbabwe, South Africa, Zambia, Nigeria, Lesotho, Sierra Leone, Swaziland, Uganda, Liberia*

√ Available policy documentation matches cost-saving intervention area
– Cost-saving intervention area not explicitly specified
X Cost-saving intervention area not mentioned in available policy documentation

GBD classification + intervention area	Intervention	Comparator	Country/ Region	Target Population	Included?	Source
**HIV/AIDS** Prevention of mother to child transmission of HIV	Pregnant women counseled, tested, and given highly active antiretroviral therapy for prevention of mother to child transmission of HIV	None	Malawi	Pregnant women	√	(3)
**Cervical cancer** HPV vaccination	HPV16/18 vaccination	None	Malawi	Females up to 12 years	√	(3)
**Malaria** Malaria vaccination	RTS,S vaccine to prevent malaria	None	Malawi	Infants 5-12 months	X	(3,4)
	RTS,S vaccine to prevent malaria	Wide-spread provision of long-lasting insecticide-treated nets (LLINs) to newborns	Malawi	Infants 5-12 months	X	(3,4)
**Malaria** LLINs for malaria prevention	Wide-spread provision of long-lasting insecticide-treated nets (LLINs) to newborns	None	Malawi	Newborns	√	(3–5)
**Syphilis** Prenatal serological screening for syphilis	New dual treponemal/nontreponemal point-of-care test (Dual-POC) for prenatal serological screening	None	Malawi + *multiple countries*	Pregnant women	√	(3,5)
	New dual treponemal/nontreponemal point-of-care test (Dual-POC) for prenatal serological screening	None	Malawi + *multiple countries*	Infants < 5	√	(3,5)
	Point-of-care treponemal immunochromatographic strip (ICS) test for prenatal serological screening	None	Malawi + *multiple countries*	Pregnant women	√	(3,5)
	Point-of-care treponemal immunochromatographic strip (ICS) test for prenatal serological screening	None	Malawi + *multiple countries*	Infants < 5	√	(3,5)

	Rapid plasma reagin plus Treponema pallidum passive particule agglutination assay for prenatal serological screening	None	Malawi + *multiple countries*	Pregnant women	√	(3,5)
	Rapid plasma reagin plus Treponema pallidum passive particule agglutination assay for prenatal serological screening	None	Malawi + *multiple countries*	Infants < 5	√	(3,5)
	Rapid plasma regain (RPR) test for prenatal serological screening	None	Malawi + *multiple countries*	Pregnant women	√	(3,5)
	Rapid plasma regain (RPR) test for prenatal serological screening	None	Malawi + *multiple countries*	Infants < 5	√	(3,5)
**HIV/AIDS** Circumcision for HIV prevention	Circumcision for prevention of heterosexual acquisition of HIV	None	Malawi + *multiple countries*	Adult males, age not specified	√	(6)
**H influenzae type B** (Hib) vaccination	Haemophilus influenzae Type b (Hib) vaccination	None	Malawi + *multiple countries*	Infants < 5	√	(3)
**Lymphatic filariasis**	Global Programme to Eliminate Lymphatic Filariasis (GPELF)	None	Malawi + *multiple countries*	All adults at risk, age not specified	X	(3)
**Sexually transmitted diseases (except HIV, cervical cancer, diarrhea)** HPV vaccination	HPV 16, 18 vaccination	None	Zimbabwe	Females aged 9	√	(7)
	Human papillomavirus (HPV) vaccine	None	Zimbabwe	Infants < 5	√	(7)
	HPV16/18 vaccination	None	Zimbabwe	Females up to 12 years	√	(7)
**HIV/AIDS** HIV self-testing	HIV self-testing	None	Zimbabwe	All persons aged 15-65	√	(8)
**Syphilis** Prenatal serological screening for syphilis	New dual treponemal/nontreponemal point-of-care test (Dual-POC) for prenatal serological screening	None	Zimbabwe + *multiple countries*	Pregnant women	–	(9)
	New dual treponemal/nontreponemal point-of-care test (Dual-POC) for prenatal serological screening	None	Zimbabwe + *multiple countries*	Infants < 5	–	(9)
	Point-of-care treponemal immunochromatographic strip (ICS)	None	Zimbabwe + *multiple countries*	Pregnant women	–	(9)

	test for prenatal serological screening					
	Point-of-care treponemal immunochromatographic strip (ICS) test for prenatal serological screening	None	Zimbabwe + *multiple countries*	Infants < 5	–	(9)
	Rapid plasma reagin plus Treponema pallidum passive particule agglutination assay for prenatal serological screening	None	Zimbabwe + *multiple countries*	Pregnant women	–	(9)
	Rapid plasma reagin plus Treponema pallidum passive particule agglutination assay for prenatal serological screening	None	Zimbabwe + *multiple countries*	Infants < 5	–	(9)
	Rapid plasma regain (RPR) test for prenatal serological screening	None	Zimbabwe + *multiple countries*	Pregnant women	–	(9)
	Rapid plasma regain (RPR) test for prenatal serological screening	None	Zimbabwe + *multiple countries*	Infants < 5	–	(9)
**HIV/AIDS** Circumcision for HIV prevention	Circumcision for prevention of heterosexual acquisition of HIV	None	Zimbabwe + *multiple countries*	Adult males, age not specified	√	(8)
**H influenzae type B** (Hib) vaccination	influenzae Type b (Hib) vaccination	None	Zimbabwe + *multiple countries*	Infants < 5	√	(7)
**HIV/AIDS** Expanded antiretroviral treatment (ART) for HIV, irrespective of CD4 count	Expanding antiretroviral treatment (ART) to CD4 count <350 + current prevention	Standard/Usual Care- Expanding antiretroviral treatment (ART) to CD4 count <200 + current prevention	South Africa	All persons aged 15-49	√	(10)
	Expanding antiretroviral treatment (ART) to CD4 count <350 + enhanced prevention	Standard/Usual Care- Expanding antiretroviral treatment (ART) to CD4 count <200 + enhanced prevention	South Africa	All persons aged 15-49	√	(10)
	Expanding antiretroviral treatment (ART) to CD4 count <500 + current prevention	Expanding antiretroviral treatment (ART) to CD4 count <350 + current prevention	South Africa	All persons aged 15-49	√	(10)

	Expanding antiretroviral treatment (ART) to CD4 count <500 + enhanced prevention	Expanding antiretroviral treatment (ART) to CD4 count <350 + enhanced prevention	South Africa	All persons aged 15-49	√	(10)
	Expanding antiretroviral treatment (ART) to all CD4 levels + current prevention	Expanding antiretroviral treatment (ART) to CD4 count <500 + current prevention	South Africa	All persons aged 15-49	√	(10)
	Expanding antiretroviral treatment (ART) to all CD4 levels + current prevention	Standard/Usual Care- Expanding antiretroviral treatment (ART) to CD4 count <200 + current prevention	South Africa	All persons aged 15-49	√	(10)
	Expanding antiretroviral treatment (ART) to all CD4 levels + enhanced prevention	Expanding antiretroviral treatment (ART) to CD4 count <500 + enhanced prevention	South Africa	All persons aged 15-49	√	(10)
	Expanding antiretroviral treatment (ART) to all CD4 levels + enhanced prevention	Standard/Usual Care- Expanding antiretroviral treatment (ART) to CD4 count <200 + enhanced prevention	South Africa	All persons aged 15-49	√	(10)
	ART initiation with CD4 count > 500	Deferred ART initiation until decline in CD4 reaches < 350, development of AIDS, or development of other conditions necessitating ART initiation	South Africa	All persons aged 19-40	√	(10)
**Cervical cancer** HPV vaccination	HPV16/18 vaccination	None	South Africa	Females up to age 12	√	(11)
**Diabetes mellitus**	Benefit-based tailored treatment	Target-to-treat	South Africa	All persons aged 20-79	–	(11)
**Tuberculosis** Bedaquiline for drug-resistant TB	Bedaquiline + background regimen	Standard/Usual Care	South Africa	All adults, age not specified	√	(10)
	Bedaquiline for extensively drug-resistant tuberculosis and standard regimen for multi-drug resistant/rifampicin-resistant tuberculosis	Standard/Usual Care	South Africa	All adults, age not specified	√	(10)

**Syphilis**<br>Prenatal serological screening for syphilis	New dual treponemal/nontreponemal point-of-care test (Dual-POC) for prenatal serological screening	None	South Africa + *multiple countries*	Pregnant women	√	(10)
	New dual treponemal/nontreponemal point-of-care test (Dual-POC) for prenatal serological screening	None	South Africa + *multiple countries*	Infants < 5	√	(10)
	Point-of-care treponemal immunochromatographic strip (ICS) test for prenatal serological screening	None	South Africa + *multiple countries*	Pregnant women	√	(10)
	Point-of-care treponemal immunochromatographic strip (ICS) test for prenatal serological screening	None	South Africa + *multiple countries*	Infants < 5	√	(10)
	Rapid plasma reagin plus Treponema pallidum passive particule agglutination assay for prenatal serological screening	None	South Africa + *multiple countries*	Pregnant women	√	(10)
	Rapid plasma reagin plus Treponema pallidum passive particule agglutination assay for prenatal serological screening	None	South Africa + *multiple countries*	Infants < 5	√	(10)
	Rapid plasma regain (RPR) test for prenatal serological screening	None	South Africa + *multiple countries*	Pregnant women	√	(10)
	Rapid plasma regain (RPR) test for prenatal serological screening	None	South Africa + *multiple countries*	Infants < 5	√	(10)
**HIV/AIDS**<br>Circumcision for HIV prevention	Circumcision for prevention of heterosexual acquisition of HIV	None	South Africa + *multiple countries*	Adult males, age not specified	√	(10)
**Cervical cancer**<br>HPV vaccination	HPV16/18 vaccination	None	Zambia	Females up to age 12	√	(12)
**HIV/AIDS**<br>Expanded antiretroviral treatment (ART) for HIV, irrespective of CD4 count	Expanded eligibility for HIV + antiretroviral therapy treatment to all HIV + patients and expanded access in routine HIV testing and linkage to care such that 80% of HIV + infected persons will receive care when eligible for treatment	Implementation of program to expand eligibility for HIV + Antiretroviral therapy treatment to patients with CD4<500 cells/ml and to expand access in routine HIV testing and linkage to	Zambia	All persons greater than or equal to age 15	√	(13)

		care such that 80% of HIV + infected persons will receive care when they are eligible for treatment				
	Expanded eligibility for HIV + antiretroviral therapy treatment to all HIV + patients and expanded access in routine HIV testing and linkage to care such that 80% of HIV + infected persons will receive care when eligible for treatment	Implementation of program to expanded access in routine HIV testing and linkage to care such that 80% of HIV + infected persons will receive care when they are eligible for treatment	Zambia	All persons greater than or equal to age 15	√	(13)
**Syphilis**<br>Prenatal serological screening for syphilis	New dual treponemal/nontreponemal point-of-care test (Dual-POC) for prenatal serological screening	None	Zambia + *multiple countries*	Pregnant women	–	(12,14,15)
	New dual treponemal/nontreponemal point-of-care test (Dual-POC) for prenatal serological screening	None	Zambia + *multiple countries*	Infants < 5	–	(12,14,15)
	Point-of-care treponemal immunochromatographic strip (ICS) test for prenatal serological screening	None	Zambia + *multiple countries*	Pregnant women	–	(12,14,15)
	Point-of-care treponemal immunochromatographic strip (ICS) test for prenatal serological screening	None	Zambia + *multiple countries*	Infants < 5	–	(12,14,15)
	Rapid plasma reagin plus Treponema pallidum passive particule agglutination assay for prenatal serological screening	None	Zambia + *multiple countries*	Pregnant women	–	(12,14,15)
	Rapid plasma reagin plus Treponema pallidum passive particule agglutination assay for prenatal serological screening	None	Zambia + *multiple countries*	Infants < 5	–	(12,14,15)
	Rapid plasma regain (RPR) test for prenatal serological screening	None	Zambia + *multiple countries*	Pregnant women	–	(12,14,15)
	Rapid plasma regain (RPR) test for prenatal serological screening	None	Zambia + *multiple countries*	Infants < 5	–	(12,14,15)

**HIV/AIDS** Circumcision for HIV prevention	Circumcision for prevention of heterosexual acquisition of HIV	None	Zambia + *multiple countries*	Adult males, age not specified	√	(12,13)
**H influenzae type B** (Hib) vaccination	Haemophilus influenzae Type b (Hib) vaccination	None	Zambia + *multiple countries*	Infants < 5	√	(14,16)
**Malaria Dihydroartemisinin piperaquine for first-line treatment**	Dihydroartemisinin piperaquine for first line treatment of plasmodium falciparum malaria (childhood malaria)	Standard/Usual Care-Artemether-Lumefantrine for first line treatment	Zambia + *multiple countries*	Infants 6-59 months	√	(17)
**Cervical cancer** HPV vaccination	HPV16/18 vaccination	None	Nigeria	Females up to age 12	–	(18–20)
**HIV/AIDS** Expanded antiretroviral treatment (ART) for HIV, irrespective of CD4 count	ART initiation with CD4 count > 500	Deferred ART initiation until decline in CD4 reaches < 350, development of AIDS, or development of other conditions necessitating ART initiation	Nigeria	All persons aged 19-40	√	(20,21)
**Syphilis** Prenatal serological screening for syphilis	New dual treponemal/nontreponemal point-of-care test (Dual-POC) for prenatal serological screening	None	Nigeria + *multiple countries*	Pregnant women	√	(19,22)
	New dual treponemal/nontreponemal point-of-care test (Dual-POC) for prenatal serological screening	None	Nigeria + *multiple countries*	Infants < 5	√	(19,22)
	Point-of-care treponemal immunochromatographic strip (ICS) test for prenatal serological screening	None	Nigeria + *multiple countries*	Pregnant women	√	(19,22)
	Point-of-care treponemal immunochromatographic strip (ICS) test for prenatal serological screening	None	Nigeria + *multiple countries*	Infants < 5	√	(19,22)
	Rapid plasma reagin plus Treponema pallidum passive particule agglutination assay for prenatal serological screening	None	Nigeria + *multiple countries*	Pregnant women	√	(19,22)
	Rapid plasma reagin plus Treponema pallidum passive particule agglutination assay for prenatal serological screening	None	Nigeria + *multiple countries*	Infants < 5	√	(19,22)

	Rapid plasma regain (RPR) test for prenatal serological screening	None	Nigeria + *multiple countries*	Pregnant women	√	(19,22)
	Rapid plasma regain (RPR) test for prenatal serological screening	None	Nigeria + *multiple countries*	Infants < 5	√	(19,22)
**HIV/AIDS** Circumcision for HIV prevention	Circumcision for prevention of heterosexual acquisition of HIV	None	Nigeria + *multiple countries*	Adult males, age not specified	√	(19,21,22)
**H influenzae type B** (Hib) vaccination	Haemophilus influenzae Type b (Hib) vaccination	None	Nigeria + *multiple countries*	Infants < 5	√	(19,22)
**Lymphatic filariasis**	Global Programme to Eliminate Lymphatic Filariasis (GPELF)	None	Nigeria + *multiple countries*	All adults at risk, age not specified	–	(19,20,22)
**Sexually transmitted diseases excluding HIV / Rotaviral enteritis** HPV vaccination	HPV 16, 18 vaccination	None	Lesotho	Females aged 9	√	(23,24)
	Human papillomavirus (HPV) vaccine	None	Lesotho	Infants < 5	√	(23,24)
**Syphilis** Prenatal serological screening for syphilis	New dual treponemal/nontreponemal point-of-care test (Dual-POC) for prenatal serological screening	None	Lesotho + *multiple countries*	Pregnant women	–	(23,25)
	New dual treponemal/nontreponemal point-of-care test (Dual-POC) for prenatal serological screening	None	Lesotho + *multiple countries*	Infants < 5	–	(23,25)
	Point-of-care treponemal immunochromatographic strip (ICS) test for prenatal serological screening	None	Lesotho + *multiple countries*	Pregnant women	–	(23,25)
	Point-of-care treponemal immunochromatographic strip (ICS) test for prenatal serological screening	None	Lesotho + *multiple countries*	Infants < 5	–	(23,25)
	Rapid plasma reagin plus Treponema pallidum passive particule agglutination assay for prenatal serological screening	None	Lesotho + *multiple countries*	Pregnant women	–	(23,25)
	Rapid plasma reagin plus Treponema pallidum passive	None	Lesotho + *multiple countries*	Infants < 5	–	(23,25)

	particule agglutination assay for prenatal serological screening					
	Rapid plasma regain (RPR) test for prenatal serological screening	None	Lesotho + *multiple countries*	Pregnant women	–	(23,25)
	Rapid plasma regain (RPR) test for prenatal serological screening	None	Lesotho + *multiple countries*	Infants < 5	–	(23,25)
**HIV/AIDS** Circumcision for HIV prevention	Circumcision for prevention of heterosexual acquisition of HIV	None	Lesotho + *multiple countries*	Adult males, age not specified	√	(26)
**H influenzae type B** (Hib) vaccination	Haemophilus influenzae Type b (Hib) vaccination	None	Lesotho + *multiple countries*	Infants < 5	√	(23)
**Cervical cancer** HPV vaccination	HPV16/18 vaccination	None	Sierra Leone	Females up to age 12	X	(27–31)
**Syphilis** Prenatal serological screening for syphilis	New dual treponemal/nontreponemal point-of-care test (Dual-POC) for prenatal serological screening	None	Sierra Leone + *multiple countries*	Pregnant women	√	(28,31)
	New dual treponemal/nontreponemal point-of-care test (Dual-POC) for prenatal serological screening	None	Sierra Leone + *multiple countries*	Infants < 5	√	(28,31)
	Point-of-care treponemal immunochromatographic strip (ICS) test for prenatal serological screening	None	Sierra Leone + *multiple countries*	Pregnant women	√	(28,31)
	Point-of-care treponemal immunochromatographic strip (ICS) test for prenatal serological screening	None	Sierra Leone + *multiple countries*	Infants < 5	√	(28,31)
	Rapid plasma reagin plus Treponema pallidum passive particule agglutination assay for prenatal serological screening	None	Sierra Leone + *multiple countries*	Pregnant women	√	(28,31)
	Rapid plasma reagin plus Treponema pallidum passive particule agglutination assay for prenatal serological screening	None	Sierra Leone + *multiple countries*	Infants < 5	√	(28,31)

	Rapid plasma regain (RPR) test for prenatal serological screening	None	Sierra Leone + *multiple countries*	Pregnant women	√	(28,31)
	Rapid plasma regain (RPR) test for prenatal serological screening	None	Sierra Leone + *multiple countries*	Infants < 5	√	(28,31)
**HIV/AIDS** Circumcision for HIV prevention	Circumcision for prevention of heterosexual acquisition of HIV	None	Sierra Leone + *multiple countries*	Adult males, age not specified	√	(32)
**H influenzae type B** (Hib) vaccination	Haemophilus influenzae Type b (Hib) vaccination	None	Sierra Leone + *multiple countries*	Infants < 5	√	(31)
**Lymphatic filariasis**	Global Programme to Eliminate Lymphatic Filariasis (GPELF)	None	Sierra Leone + *multiple countries*	All adults at risk, age not specified	√	(27,29,30,33)
**Cervical cancer** HPV vaccination	HPV16/18 vaccination	None	Swaziland	Females up to age 12	√	(34)
**Syphilis** Prenatal serological screening for syphilis	New dual treponemal/nontreponemal point-of-care test (Dual-POC) for prenatal serological screening	None	Swaziland + *multiple countries*	Pregnant women	X	(34–36)
	New dual treponemal/nontreponemal point-of-care test (Dual-POC) for prenatal serological screening	None	Swaziland + *multiple countries*	Infants < 5	X	(34–36)
	Point-of-care treponemal immunochromatographic strip (ICS) test for prenatal serological screening	None	Swaziland + *multiple countries*	Pregnant women	X	(34–36)
	Point-of-care treponemal immunochromatographic strip (ICS) test for prenatal serological screening	None	Swaziland + *multiple countries*	Infants < 5	X	(34–36)
	Rapid plasma reagin plus Treponema pallidum passive particule agglutination assay for prenatal serological screening	None	Swaziland + *multiple countries*	Pregnant women	X	(34–36)

	Rapid plasma reagin plus Treponema pallidum passive particule agglutination assay for prenatal serological screening	None	Swaziland + *multiple countries*	Infants < 5	X	(34–36)
	Rapid plasma regain (RPR) test for prenatal serological screening	None	Swaziland + *multiple countries*	Pregnant women	X	(34–36)
	Rapid plasma regain (RPR) test for prenatal serological screening	None	Swaziland + *multiple countries*	Infants < 5	X	(34–36)
**HIV/AIDS** Circumcision for HIV prevention	Circumcision for prevention of heterosexual acquisition of HIV	None	Swaziland + *multiple countries*	Adult males, age not specified	√	(37,38)
**Sexually transmitted diseases (except HIV, cervical cancer, diarrhea)** HPV vaccination	HPV16/18 vaccination	None	Cameroon	Females up to age 12	X	(39)
**Syphilis** Prenatal serological screening for syphilis	New dual treponemal/nontreponemal point-of-care test (Dual-POC) for prenatal serological screening	None	Cameroon + *multiple countries*	Pregnant women	X	(39)
	New dual treponemal/nontreponemal point-of-care test (Dual-POC) for prenatal serological screening	None	Cameroon + *multiple countries*	Infants < 5	X	(39)
	Point-of-care treponemal immunochromatographic strip (ICS) test for prenatal serological screening	None	Cameroon + *multiple countries*	Pregnant women	X	(39)
	Point-of-care treponemal immunochromatographic strip (ICS) test for prenatal serological screening	None	Cameroon + *multiple countries*	Infants < 5	X	(39)
	Rapid plasma reagin plus Treponema pallidum passive particule agglutination assay for prenatal serological screening	None	Cameroon + *multiple countries*	Pregnant women	X	(39)
	Rapid plasma reagin plus Treponema pallidum passive particule agglutination assay for prenatal serological screening	None	Cameroon + *multiple countries*	Infants < 5	X	(39)

	Rapid plasma regain (RPR) test for prenatal serological screening	None	Cameroon + *multiple countries*	Pregnant women	X	(39)
	Rapid plasma regain (RPR) test for prenatal serological screening	None	Cameroon + *multiple countries*	Infants < 5	X	(39)
**HIV/AIDS** Circumcision for HIV prevention	Circumcision for prevention of heterosexual acquisition of HIV	None	Cameroon + *multiple countries*	Adult males, age not specified	X	(39,40)
**H influenzae type B** (Hib) vaccination	Haemophilus influenzae Type b (Hib) vaccination	None	Cameroon + *multiple countries*	Infants < 5	√	(39)
**Lymphatic filariasis**	Global Programme to Eliminate Lymphatic Filariasis (GPELF)	None	Cameroon + *multiple countries*	All adults at risk, age not specified	√	(39)
**Cervical cancer** HPV vaccination	HPV16/18 vaccination	None	Liberia	Females up to age 12	√	(41)
**Syphilis** Prenatal serological screening for syphilis	New dual treponemal/nontreponemal point-of-care test (Dual-POC) for prenatal serological screening	None	Liberia + *multiple countries*	Pregnant women	–	(42)
	New dual treponemal/nontreponemal point-of-care test (Dual-POC) for prenatal serological screening	None	Liberia + *multiple countries*	Infants < 5	–	(42)
	Point-of-care treponemal immunochromatographic strip (ICS) test for prenatal serological screening	None	Liberia + *multiple countries*	Pregnant women	–	(42)
	Point-of-care treponemal immunochromatographic strip (ICS) test for prenatal serological screening	None	Liberia + *multiple countries*	Infants < 5	–	(42)
	Rapid plasma reagin plus Treponema pallidum passive particule agglutination assay for prenatal serological screening	None	Liberia + *multiple countries*	Pregnant women	–	(42)
	Rapid plasma reagin plus Treponema pallidum passive particule agglutination assay for prenatal serological screening	None	Liberia + *multiple countries*	Infants < 5	–	(42)

	Rapid plasma regain (RPR) test for prenatal serological screening	None	Liberia + *multiple countries*	Pregnant women	–	(42)
	Rapid plasma regain (RPR) test for prenatal serological screening	None	Liberia + *multiple countries*	Infants < 5	–	(42)
**HIV/AIDS** Circumcision for HIV prevention	Circumcision for prevention of heterosexual acquisition of HIV	None	Liberia + *multiple countries*	Adult males, age not specified	√	(43)
**H influenzae type B** (Hib) vaccination	Haemophilus influenzae Type b (Hib) vaccination	None	Liberia + *multiple countries*	Infants < 5	√	(41,42,44)
**Lymphatic filariasis**	Global Programme to Eliminate Lymphatic Filariasis (GPELF)	None	Liberia + *multiple countries*	All adults at risk, age not specified	–	(42,44)

* Time spans of each included plan: Malawi: 2015-2022; Zimbabwe: 2015-2020; South Africa: 2015-2022; Zambia: 2015-2021; Nigeria: 2010-2018; Lesotho: 2012-2022; Sierra Leone: 2010-2021; Swaziland: 2014-2018; Cameroon: 2016-2020; Liberia: 2011-2021

Appendix, Table 3: Excluded countries in our sample identification DALYs/100,000 extracted from 2017 (45)		
**Country**	**DALYs per 100,000**	**Reason for exclusion**
Central African Republic	89,692	Official languages: French and Sango + no cost-saving interventions published
South Sudan	71,068	No cost-saving interventions published
Chad	71,486	Official languages: Arabic and French + no cost-saving interventions published
Mali	69,096	Official language: French + no cost-saving interventions published
Niger	61,357	Official language: French + no cost-saving interventions published
Burkina Faso	61,147	Official languages: French, Moore, Fula, Dioula
Guinea	58,109	Official language: French
Mozambique	56,427	Official language: Portuguese
Somalia	58,321	No cost-saving interventions published
Guinea Bissau	55,152	Official languages: Portuguese and Upper Guinea Creole
Democratic Republic of Congo	54,769	Official languages: French, Lingala, Kikongo, Swahili, Tshiluba + no cost-saving interventions published
Papua New Guinea	52,604	No cost-saving interventions published
Burundi	50,800	Official languages: Kirundi, French, and English; Policy documentation in French
Côte d'Ivoire	50,387	Official language: French + no cost-saving interventions published
Madagascar	50,125	Official languages: Malagasy and French
Republic of Congo	45,188	Official languages: French, Kituba, Kikongo, Lingala
Angola	46,503	Official language: Portuguese

APPENDIX REFERENCES

1. Neumann PJ, Thorat T, Zhong Y, Anderson J, Farquhar M, Salem M, et al. A Systematic Review of Cost-Effectiveness Studies Reporting Cost-per-DALY Averted. Speybroeck N, editor. PLOS ONE. 2016 Dec 22;11(12):e0168512.

2. Neumann PJ, Greenberg D, Olchanski NV, Stone PW, Rosen AB. Growth and Quality of the Cost–Utility Literature, 1976–2001. Value Health. 2005 Jan 1;8(1):3–9.

3. Malawi Ministry of Health. Malawi National Community Health Strategy 2017 - 2022 [Internet]. Healthy Newborn Network. [cited 2018 Nov 26]. Available from: https://www.healthynewbornnetwork.org/resource/malawi-national-community-health-strategy-2017-2022/

4. USAID, HHS, CDC, US Department of State. US President's Malaria Initiative, Malawi: Abbreviated Malaria Operational Plan FY 2019 [Internet]. Available from: https://www.pmi.gov/docs/default-source/default-document-library/malaria-operational-plans/fy19/fy-2019-malawi-abbreviated-malaria-operational-plan.pdf?sfvrsn=5

5. Health Finance and Governance Project, USAID. Essential Health Services: Malawi | HFG [Internet]. 2015 [cited 2018 Nov 26]. Available from: https://www.hfgproject.org/essential-package-of-health-services-country-snapshot-malawi/

6. PEPFAR & Government of Malawi. Malawi Country Operational Plan 2018: Strategic Direction Summary [Internet]. 2017. Available from: https://www.pepfar.gov/documents/organization/285859.pdf

7. Zimbabwe Ministry of Health and Child Care. Zimbabwe Expanded Programme on Immunisation Comprehensive Multi Year Plan (2015-2019) [Internet]. Available from: http://www.nationalplanningcycles.org/sites/default/files/country_docs/Zimbabwe/zimbabwe_epi_cmyp_2015_-_2019.pdf

8. PEPFAR & Government of Zimbabwe. Zimbabwe Country Operational Plan 2018: Revised Strategic Direction Summary [Internet]. Available from: https://www.pepfar.gov/documents/organization/285847.pdf

9. Zimbabwe Ministry of Health and Child Care. The National Health Strategy for Zimbabwe (2016-2020). Equity & Quality in Health: Leaving No One Behind. [Internet]. Available from: https://www.unicef.org/zimbabwe/National_Health_Strategy_for_Zimbabwe_2016-2020_FINAL.pdf

10. South Africa National Department of Health. South Africa's National Strategic Plan for HIV, TB, and STIs (2017-2022) [Internet]. Available from: http://sanac.org.za/wp-content/uploads/2017/05/NSP_FullDocument_FINAL.pdf

11. South Africa National Department of Health. South Africa's Department of Health National Strategic Plan (2015-2020) [Internet]. Available from: https://www.childrenandaids.org/sites/default/files/2017-04/SouthAfrica_National-Health-Strategic-Plan_2015.pdf

12. Zambia Ministry of Health. Zambia National Health Strategic Plan 2017-2021. 2016 [cited 2018 Nov 26]; Available from: http://dspace.unza.zm:8080/xmlui/handle/123456789/5113

13. PEPFAR & Government of Zambia. Zambia Country Operational Plan 2018: Strategic Direction Summary [Internet]. 2018. Available from: https://www.pepfar.gov/documents/organization/285848.pdf

14. Health Finance and Governance Project, USAID. Essential Health Services: Zambia | HFG [Internet]. 2015 [cited 2018 Nov 26]. Available from: https://www.hfgproject.org/essential-package-of-health-services-country-snapshot-zambia/

15. Zambia Ministry of Health. Zambia's Reproductive, Maternal, Newborn, Child, and Adolescent Health and Nutrition Communication and Advocacy Strategy 2018-2021 [Internet]. Available from: http://www.moh.gov.zm/?wpfb_dl=111

16. African Health Observatory [Internet]. African Health Observatory. [cited 2018 Dec 17]. Available from: http://www.aho.afro.who.int/en

17. USAID, HHS, CDC, US Department of State. US President's Malaria Initiative, Zambia: Malaria Operational Plan FY 2019 [Internet]. Available from: https://www.pmi.gov/docs/default-source/default-document-library/malaria-operational-plans/fy19/fy-2019-zambia-malaria-operational-plan.pdf?sfvrsn=3

18. Nigeria National Primary Health Care Development Agency. Nigerian National Routine Immunization Strategic Plan (2013-2015) [Internet]. Available from: http://www.nationalplanningcycles.org/sites/default/files/country_docs/Nigeria/ri_strategic_plan_combined_mahmud_draft_1.pdf

19. Health Finance and Governance Project, USAID. Essential Health Services: Nigeria | HFG [Internet]. [cited 2018 Dec 17]. Available from: https://www.hfgproject.org/essential-package-of-health-services-country-snapshot-nigeria/

20. Nigeria Federal Ministry of Health. Nigeria National Health Policy 2016: Promoting the Health of Nigerians to Accelerate Socio-Economic Development [Internet]. 2016. Available from: http://www.nationalplanningcycles.org/sites/default/files/planning_cycle_repository/nigeria/draft_nigeria_national_health_policy_final_december_fmoh_edited.pdf

21. PEPFAR & Government of Nigeria. Nigeria Country Operational Plan 2018: Strategic Direction Summary [Internet]. 2018. Available from: https://www.pepfar.gov/documents/organization/285856.pdf

22. Federal Ministry of Health. Nigeria National Strategic Health Development Plan (NSHDP) 2010-2015.

23. Government of Lesotho. Lesotho National Health Strategic Plan (NHSP 2017-2022) [Internet]. 2016. Available from: http://www.nationalplanningcycles.org/sites/default/files/planning_cycle_repository/lesotho/lesotho_revised_nhsp_2017-22_final_draft1.pdf

24. Government of Lesotho. Lesotho Expanded Programme on Immunisation Comprehensive Multi-Year pLan (2012-2016) [Internet]. 2012. Available from: http://www.nationalplanningcycles.org/sites/default/files/country_docs/Lesotho/new_cmulti_year_plan_2012-2016_for_submission_revised_on_17_jan_2012.pdf

25. Government of Lesotho. Lesotho Health Sector Strategic Plan 2012-2017. 2013.

26. PEPFAR & Governmet of Lesotho. Lesotho Country Operational Plan 2018: Strategic Direction Summary [Internet]. 2018. Available from: https://www.pepfar.gov/documents/organization/285860.pdf

27. Ministry of Health and Sanitation. Sierra Leone Annual Health Sector Performance Report 2016 [Internet]. Available from: https://afro.who.int/sites/default/files/2017-08/Sierra%20Leone%20Health%20Sector%20%20Performance%20Report%202016.pdf

28. Ministry of Health and Sanitation. Sierra Leone Basic Package of Essential Health Services [Internet]. 2010. Available from: http://apps.who.int/medicinedocs/documents/s19423en/s19423en.pdf

29. Ministry of Health and Sanitation, Health Education Division. Sierra Leone National Health Promotion Strategy 2017-2021. 2016.

30. Ministry of Health and Sanitation. Sierra Leone National Health Sector Strategic Plan 2017-2021 [Internet]. 2017. Available from: http://www.nationalplanningcycles.org/sites/default/files/planning_cycle_repository/sierra_leone/sierra_leone_nhssp_2017-21_final_sept2017.pdf

31. Ministry of Health and Sanitation. Sierra Leone Basic Package of Essential Health Services 2015-2020 [Internet]. 2015. Available from: https://mohs2017.files.wordpress.com/2017/06/gosl_2015_basic-package-of-essential-health-services-2015-2020.pdf

32. Sierra Leone National AIDS Secretariat. Sierra Leone National Strategic Plan on HIV/AIDS 2016-2020: Rapid Response Initiative to Ending HIV as a Public Health and Development Problem. 2015.

33. Sierra Leone Ministry of Health and Sanitation. Sierra Leone National Neglected Tropical Disease Programme (NTDP): Master Plan for Neglected Tropical Diseases Elimination in Sierra Leone (2016-2020).

34. Swaziland - The Second National Health Sector Strategic Plan 2014-2018: Towards attainment of Universal Health Coverage [Internet]. 2014. Available from: http://www.nationalplanningcycles.org/sites/default/files/planning_cycle_repository/swaziland/swaziland_nhssp_ii_draft_zero_29_aug_2014.pdf

35. Swaziland Ministry of Health. Swaziland National Policy on Sexual and Reproductive Health | K4Health [Internet]. [cited 2018 Nov 26]. Available from: https://www.k4health.org/toolkits/swaziland-hiv-prevention/swaziland-national-policy-sexual-and-reproductive-health

36. Kingdom of Swaziland. Swaziland’s Government Programme of Action 2013-2018, Ministries’ Action Plans to 2018 and 2022. [Internet]. Available from: http://www.gov.sz/images/ministries%20action%20plans%20pdf.pdf

37. Swaziland Ministry of Health. Swaziland Male Circumcision Strategic and Operational Plan for HIV Prevention 2014-2018 [Internet]. Available from: https://www.infocenter.nercha.org.sz/sites/default/files/Swaziland%20Male%20Circumcision%20Strategic%20and%20Operational%20Plan%202014%20-2018.pdf

38. PEPFAR & Government of Swaziland. Swaziland Country Operational Plan 2018: Strategic Direction Summary [Internet]. 2018. Available from: https://www.pepfar.gov/documents/organization/285864.pdf

39. Ministry of Public Health. Cameroon National Health Development Plan 2016-2020 [Internet]. 2016. Available from: https://www.minsante.cm/site/?q=en/content/national-health-development-plan-nhdp-2016-2020

40. PEPFAR & Government of Cameroon. Cameroon Country Operational Plan 2019: Strategic Direction Summary [Internet]. 2019. Available from: https://www.state.gov/wp-content/uploads/2019/09/Cameroon_COP19-Strategic-Directional-Summary_public.pdf

41. Liberia Ministry of Health and Social Welfare. Liberia Ministry of Health National EPI Strategic Plan (Draft) 2016-2020 [Internet]. Available from: http://www.nationalplanningcycles.org/sites/default/files/planning_cycle_repository/liberia/liberia_cmyp_2016_2020.pdf

42. Liberia Ministry of Health and Social Welfare. Liberia National Health & Social Welfare Policy and Plan 2011-2021 [Internet]. Available from: http://moh.gov.lr/wp-content/uploads/2017/03/2011-2021-National-Health-Policy-Plan_MOH.pdf

43. Liberia National AIDS Commission. Liberia National HIV Prevention Strategy 2017-2020 [Internet]. Available from: http://www.nacliberia.org/doc/HIV%20prevention%20strategy%20Liberia-_Final%20Version_%2018-04-17_.pdf

44. Liberia Ministry of Health and Social Welfare. Liberia National Policy & Strategic Plan on Health Promotion 2016-2021 [Internet]. Available from: https://afro.who.int/sites/default/files/2017-06/National%20Policy%20and%20Strategic%20Plan%20on%20Health%20Promotion%2C%20Liberia%2C%202016%20-%202021.pdf

45. IHME. GBD Compare | IHME Viz Hub [Internet]. [cited 2018 Nov 16]. Available from: http://vizhub.healthdata.org/gbd-compare
